# Decreased CSF clearance and increased brain amyloid in Alzheimer’s disease

**DOI:** 10.1186/s12987-022-00318-y

**Published:** 2022-03-14

**Authors:** Yi Li, Henry Rusinek, Tracy Butler, Lidia Glodzik, Elizabeth Pirraglia, John Babich, P. David Mozley, Sadek Nehmeh, Silky Pahlajani, Xiuyuan Wang, Emily B. Tanzi, Liangdong Zhou, Sara Strauss, Roxana O. Carare, Neil Theise, Nobuyuki Okamura, Mony J. de Leon

**Affiliations:** 1 Department of Radiology, Weill Cornell Medicine, Cornell University, Brain Health Imaging Institute, 407 East 61 Street, New York, NY 10021 USA; 2 Department of Radiology, New York University School of Medicine, New York, NY USA; 3 Division of Pharmacology, Faculty of Medicine, Tohoku Medical and Pharmaceutical University, Sendai, Japan; 4 Department of Psychiatry, New York University School of Medicine, New York, NY USA; 5 Department of Clinical Neuroanatomy, University of Southampton, Southampton, UK; 6 Department of Pathology, New York University School of Medicine, New York, NY USA

## Abstract

**Background:**

In sporadic Alzheimer’s disease (AD), brain amyloid-beta (Aβ) deposition is believed to be a consequence of impaired Aβ clearance, but this relationship is not well established in living humans. CSF clearance, a major feature of brain glymphatic clearance (BGC), has been shown to be abnormal in AD murine models. MRI phase contrast and intrathecally delivered contrast studies have reported reduced CSF flow in AD. Using PET and tau tracer ^18^F-THK5117, we previously reported that the ventricular CSF clearance of the PET tracer was reduced in AD and associated with elevated brain Aβ levels.

**Methods:**

In the present study, we use two PET tracers, ^18^F-THK5351 and ^11^C-PiB to estimate CSF clearance calculated from early dynamic PET frames in 9 normal controls and 15 AD participants.

**Results:**

we observed that the ventricular CSF clearance measures were correlated (r = 0.66, p < 0.01), with reductions in AD of 18 and 27%, respectively. We also replicated a significant relationship between ventricular CSF clearance (^18^F-THK5351) and brain Aβ load (r =  − 0.64, n = 24, p < 0.01). With a larger sample size, we extended our observations to show that reduced CSF clearance is associated with reductions in cortical thickness and cognitive performance.

**Conclusions:**

Overall, the findings support the hypothesis that failed CSF clearance is a feature of AD that is related to Aβ deposition and to the pathology of AD. Longitudinal studies are needed to determine whether failed CSF clearance is a predictor of progressive amyloidosis or its consequence.

## Introduction

Two decades after it was hypothesized that the amyloidosis of sporadic Alzheimer's disease (AD) is mechanistically related to the impaired clearance of Aβ from the parenchyma [1, 2], there is limited understanding of the pathophysiology of brain Aβ clearance in sporadic AD [3–5]. Part of the difficulty relates to the absence of direct, non-invasive methods to examine the role of human CSF as a carrier of Aβ and other waste molecules. Human lumbar spine CSF sampling studies with ^13^C_6_-leucine have demonstrated a deficit in the clearance of Aβ from the CNS to the periphery [6]. Murine studies have revealed a brain glymphatic clearance system where waste products including Aβ are carried in the interstitial fluid (ISF) and CSF [7–9] and drain into dural lymphatics that contribute to immune surveillance [10]. In transgenic AD models, these clearance deficits are progressive [11].

Current reviews have highlighted the recent interest in neuroimaging using Magnetic Resonance Imaging (MRI) and Positron Emission Tomography (PET) to investigate the glymphatic transport function in the live animal and human brain [12–15]. We observed using ^18^F-THK5117 that ventricular CSF clearance was reduced in AD and inversely related to the magnitude of brain Aβ deposits measured with ^11^C-PiB [16]. Several confirmatory PET quantification studies of CSF clearance were subsequently reported [17, 18]. The present PET study uses ^18^F-THK5351 dynamics to measure CSF clearance, (11)C-labeled Pittsburgh Compound-B (^11^C-PiB) uptake to measure the amyloid burden, and T1-weighted MRI to estimate brain atrophy in mild AD and healthy elderly participants. Our results with ^18^F-THK5351 radiotracer replicate the AD clearance reductions as well as the association of the clearance deficit with the Aβ lesion burden seen with ^18^F-THK5117. We now observe for the first time that the magnitude of CSF clearance is inversely related to decreased cortical thickness and to decreased cognitive function.

## Materials and methods

### Study participants

24 older adults participants (age range 61–90 y, 10 male and 14 female) participated in this IRB approved study. Written informed consent was obtained from each participant or their legal caretakers. Participants included 9 normal controls (NL) and 15 AD participants including 6 very mild cases (CDR = 0.5) and 9 with mild to moderate cognitive symptoms (CDR = 1.0) [19, 20]. All participants received standardized clinical and neuropsychological assessments, including Clinical Dementia Rating (CDR) Scale, Mini-Mental State Exam (MMSE) and Alzheimer's Disease Assessment Scale–Cognitive Subscale (ADAS-cog). All AD participants showed amyloid deposits in cerebral cortex, with global PiB uptake ratio greater than 1.25 [19]. AD patients were recruited from the memory clinic of Tohoku University Hospital. Control subjects were recruited from the general community. Subject’s diagnoses were made at a consensus conference according to the National Institute of Neurological and Communicative Disorders and Stroke/AD and Related Disorders Association criteria [20].

### PET and MRI image acquisition

Each study participant received within a 3-month interval, the clinical assessments, a high resolution T1-weighted MRI, and two dynamic PET exams, one exam performed with ^18^F-THK5351 tracer used to estimate CSF clearance and the other with ^11^C-PiB- for Aβ. The syntheses of ^18^F-THK5351 and ^11^C-PiB compounds were previously described [21, 22]. PET imaging was performed using an Eminence STARGATE PET-CT scanner (Shimadzu, Kyoto, Japan). Intravenous injections of ^18^F-THK5351 (185 MBq) or ^11^C-PiB (296 MBq) took place on two separate days. Dynamic PET data were obtained in list mode for 60 min for ^18^F-THK5351 and 70 min for ^11^C-PiB. Images were reconstructed to a 128 × 128 × 79 matrix of 2 × 2 × 2.6 mm voxels in 26 time frames for ^18^F-THK5351 and 25 frames for ^11^C-PiB. MR images were obtained using a SIGNA 1.5 T unit (General Electric, Milwaukee, WI). The MRI protocol included a 3D volumetric acquisition of a T1-weighted gradient echo sequence with parameters: echo time/repetition time 2.4/50 ms; flip angle 45°; acquisition matrix 256 × 256; 1 excitation; field of view 22 cm; and a 2.0 mm slice thickness without gaps.

### MRI segmentation

FreeSurfer software (v. 6.1) was used for MRI brain segmentation [23]. Regions of Interest (ROIs) were determined for the neocortical gray matter (GM) and white matter (WM), the cerebellar gray matter, and the lateral ventricle [24]. The whole brain ROI was derived from FreeSurfer aparc + aseg file [25] which includes the complete cerebral and cerebellar volume and pons. The average thickness of the neocortical GM was used as an index of brain atrophy. Statistical Parametric Mapping (SPM 12) software (www.fil.ion.ucl.ac.uk/spm) was used to calculate the total intracranial volumes. To optimize CSF sampling from the lateral ventricle and minimize partial volume contamination by brain tissue, an eroded lateral ventricle mask (ELVM) was created using the 4 mm 3D erosion of MRI-segmented ventricles. Both ^18^F-THK5351 and ^11^C-PiB scans have been visually examined, and no choroid plexus binding was observed.

### PET image workflow

After decay correction, standardized uptake value (SUV) time-framed images were created by normalizing the reconstructed radioactivity by injected dose and body weight. To minimize the effect of head motion for each subject, the dynamic PET frames were realigned to the first-time frame using SPM. The anatomically segmented MR images were co-registered separately to the space of the ^18^F-THK5351 and ^11^C-PiB PET scans using SPM12, and the native PET data weren sampled. Satisfactory inter-modality alignment was verified for each exam by an experienced neuroradiologist. Each PET time frame was partial volume corrected (PVC) using a modified one tissue model [26].

### Estimations of PET tracer clearance

**Fig. 1:**
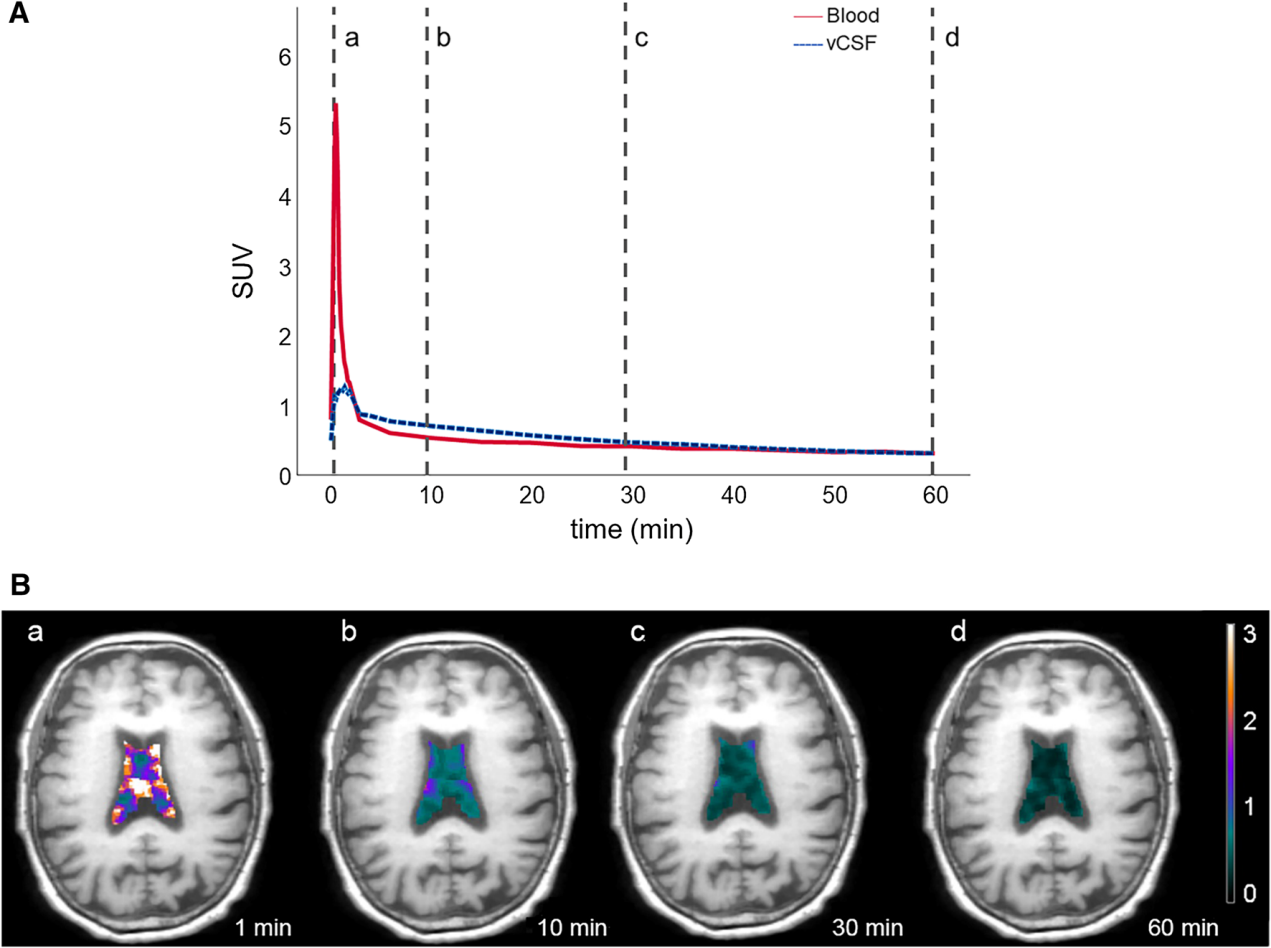
**A** The ^18^F-THK5351Ventricular CSF(t) time activity curve (TAC) and the image-derived arterial blood curve, averaged over all subjects. The tracer TAC peaked approximately within 2 min after injection. The lines marked a, b, c, d correspond to the sampled time points for vCSF-SLOPE and to the images of the four time frames shown in (**B**). standardized uptake value (SUV). **B** The four images from one AD subject demonstrate the changes in ^18^F-THK5351 SUV in the lateral ventricle superimposed on the coregistered MRI. Color maps and the corresponding color bar (SUVR) depict the decreasing CSF levels of the tracer at 1, 10, 30 and 60 min

Ventricular CSF, whole brain and blood time activity curves (TAC) were derived for each participant. Tracer concentrations in the blood were sampled from the internal carotid artery, guided by MR scans that were co-registered to the PET data [27]. Blood TAC reached a peak within 2 min and approached low asymptotic time course by 4 min (Fig. 1a). Brain TACs reached a peak between 2 and 4 min after injection. The ventricular CSF clearance was derived from the slope of a linear regression fit of SUV(t) for ELVM (see “MRI segmentation” Section) over 10–30 min [28]. The 10–30 min time frame was selected to minimize the potential contribution of blood in the choroid plexus. To control for the variability in the amount of radiotracer delivered to the brain across participants, the slope was normalized by the total brain SUV of the tracer over the first 4 min after injection. All slopes were negative and for calculations the absolute slope value was used to represent the clearance magnitude. The resulting normalized absolute value slope was denoted as vCSF-SLOPE and used throughout this study to estimate CSF clearance rate.

### PET SUVr estimations of brain amyloid

The amyloid binding of cerebral grey matter was estimated using ^11^C-PiB binding based on the 50–70 min interval, as we reported previously [29]. The same uptake period for the cerebellar gray matter was used as the reference tissue in the standardized uptake value ratio (SUVr).

### Statistical analysis

The general linear model and univariate analysis of variance (ANOVA), with Tukey post hoc tests, were used to examine the vCSF-SLOPE across the NL and AD clinical groups. All significant results were confirmed using the nonparametric Mann–Whitney test with Bonferroni corrections for multiple comparisons. The relationships between the vCSF-SLOPE and Aβ binding, cortical thickness and cognitive function were tested using parametric correlation models, and nonparametric correlation models were tested if measures were not normally distributed. The ventricular volume and age were examined as covariates in the regression models. All statistical tests were two-sided and significance was set at p < 0.05. All analyses were checked for violations of the model assumptions, and no conflicts were found.

## Results

### Clinical data

The demographic data from the NL and AD groups are shown in Table 1. There were no age or gender group differences. The ADAS-cog and MMSE were significantly different in AD participants as compared with NL (ADAS-cog: F = 21.1 p < 0.01, MMSE: F = 23.5 p < 0.01).

**Table 1:** Participant demographic data

	N	age	Gender(M/F)	MMSE*	ADAS-cog*
NL	9	72.8 (8.9)	3/6	28.8 (1.3)	4.8 (2.2)
AD	15	76.3 (8.9)	7/8	21.9 (4.3)	16.5 (7.1)

Values are expressed as mean (SD)

*p < 0.0.01, between NL and AD groups

### Clearance derived from ^18^F-THK5351 and ^11^C-PiB tracers

#### Group clearance effects

The ^18^F-THK5351 and ^11^C-PiB tracers showed very similar and significantly lower vCSF-SLOPE in AD as compared with NL, see Table 2. The vCSF-SLOPE_THK5351_ was reduced in AD by 20% (F = 10.2, p < 0.01). The vCSF-SLOPE_PiB_ was reduced by 28% in AD (F = 24.4, p < 0.01,). The findings remained significant after PVC (both p’s < 0.01).

**Table 2:** vCSF-SLOPE and tracer binding

Group	vCSF-SLOPE_THK5351_	vCSF-SLOPE_PiB_	^11^C-PiB GM SUVr 50–70 min	^11^C-PiB GM SUVr 10–30 min	^18^F-THK5351 GM SUVr 10–30 min	^18^F-THK5351 GM SUVr 10–30 min
NL	0.11 (0.02)	0.11 (0.01)	1.28 (0.17)	1.02 (0.05)	1.30 (0.12)	1.41 (0.20)
AD	0.09 (0.02)	0.08 (0.02)	1.88 (0.30)	1.25 (0.12)	1.37 (0.10)	1.63 (0.11)
Diff. from normal	− 18%, p < 0.01	− 27%, p < 0.01	46%, p < 0.01	22%, p < 0.01	5%, NS	16%, p < 0.05

SUVr: standardized uptake value ratio

p-value less than 0.05 (≤ 0.05) is statistically significant

#### Within subject vCSF

**Fig. 2:**
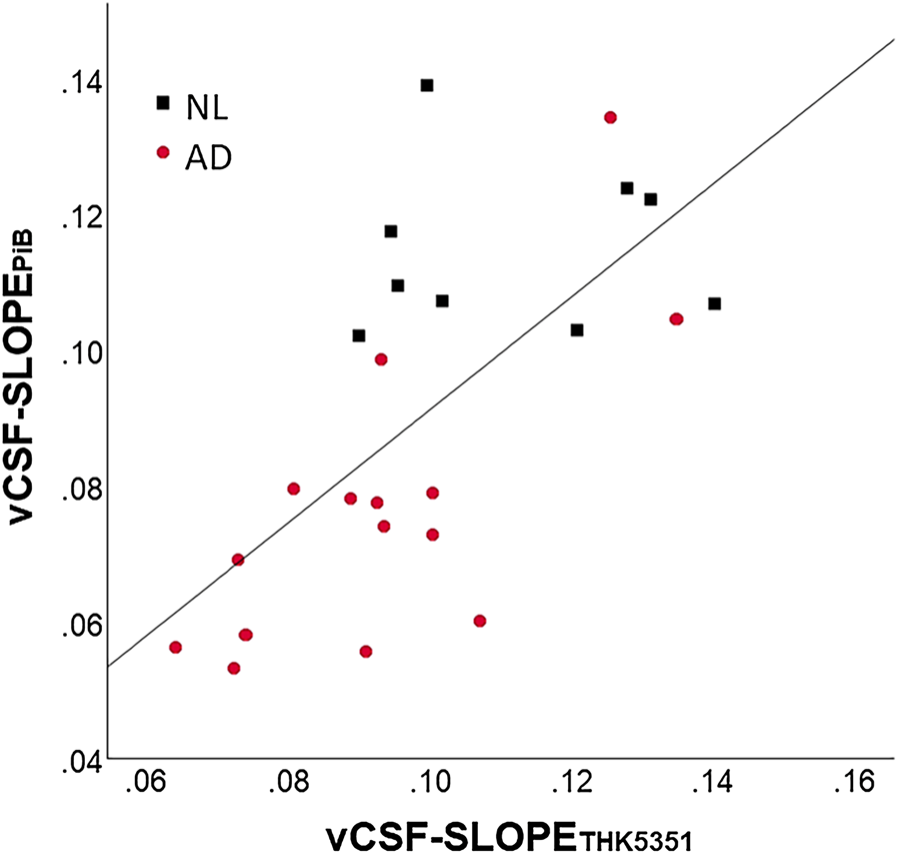
Cross-tracer agreement of clearance: the vCSF-SLOPE for ^11^C-PiB and ^18^F-THK5351 PET tracers are highly correlated (r = 0.66, n = 24, p < 0.01)

Across the entire sample, the vCSF-SLOPE_**THK5351**_ and vCSF-SLOPE_**PiB**_ were closely associated (r = 0.66, p < 0.01, n = 24, see Fig. 2). The correlation remained significant when the analysis was restricted to the AD group (r = 0.61, p < 0.05, n = 15).

#### ^11^C-PiB and ^18^F-THK5351 gray matter binding

The PiB PET examined with the 50–70 min gray matter SUVr showed significantly higher (46%) GM binding in AD as compared with NL (see Table 2). We also tested both tracers for gray matter binding during the 10–30 min interval when clearance measurements were made. For the PiB-PET the SUVr binding effect was significantly higher in the AD group (22%, p < 0.01) than in NL. However, for THK5331-PET the SUVr was only 5% higher in AD vs NL and this effect was not significant (p > 0.05).

#### The relationship between vCSF clearance and amyloid binding

**Fig. 3:**
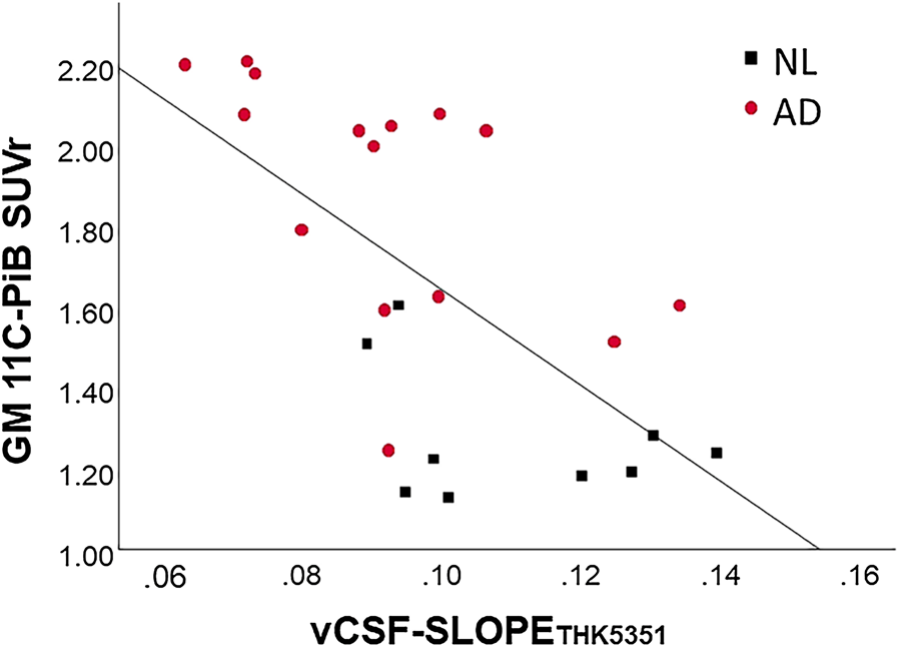
The vCSF-SLOPE_THK5351_ is inversly correlated with the extent of fibrillar Aβ as estimated by ^11^C-PiB gray matter binding (r =  − 0.64, p < 0.01, n = 24). The correlation remains significant when restricted to the AD group (r =  − 0.58, p < 0.05, n = 15). NL: (black); AD: (red)

Across all participants, the vCSF-SLOPE_THK5351_ was inversely correlated with the Aβ GM binding (r = -0.64, p < 0.01, n = 24, see Fig. 3). This correlation remained significant after PVC (r = − 0.72, n = 24, p < 0.01). Importantly, the relationship between vCSF-SLOPE and Aβ GM binding remained significant when restricted to the AD group (r =  −  0.58, n = 15, p < 0.05). The relationship between vCSF-SLOPE and GM Aβ binding was not significant in the NL group (p > 0.05), presumably due to a narrow range of Aβ binding in this group.

#### The relationship of CSF clearance and cortical thickness

The vCSF-SLOPE_THK5351_ was positively correlated with the cerebral cortex thickness (r = 0.55, n = 24, p < 0.01). The correlation was a trend when restricted to the AD group (r = 0.45, n = 15, p = 0.08), reaching p < 0.05 significance in a one-tailed test. However, it was not significant in the NL group.

#### CSF clearance, ventricular volume and age

To rule out ventricular size and age as confounds for the vCSF-SLOPE measure, we tested the ventricular volume and age as covariates in our regression models. We observed that for the total group the correlation between vCSF-SLOPE_THK5331_ and Aβ GM binding remained significant after adjustment for age and ventricular size (r =  −  0.44, p < 0.05, n = 24). In an analysis restricted to the AD group, this relationship also remained significant even after controlling for age and ventricular size (r = − 0.56, n = 15, p < 0.05).

#### CSF clearance vs tracer binding and cognitive performance

Over all participants, the vCSF clearance was inversely correlated with the ADAS-cog (vCSF-SLOPE_THK5351_ r = − 0.55, p < 0.01, n = 24). Studying the clinical groups separately showed that only in the NL group did the association of vCSF-SLOPE_THK5351_ and ADAS-cog remain significant (r = − 0.83, n = 9, p < 0.01). Over all participants, the PiB tracer binding in GM was also correlated with the ADAS-cog (r = 0.77, p < 0.01, n = 24). Studying the two groups separately showed that only in the AD group did the association of the PiB tracer GM binding and ADAS-cog remain significant (r = 0.58, n = 15, p < 0.05).

## Discussion

### The impaired clearance of CSF in AD

The impaired clearance of the CSF and interstitial fluid (ISF) are thought to play a role in the deposition of Aβ in the AD brain [1, 30–32]. Abnormalities in several physiological clearance mechanisms that potentially underlie Aβ removal from the brain have been shown in animal models. Recent transgenic AD mouse studies have demonstrated age-related reductions in ISF and CSF clearance as well as CSF clearance deficits prior to Aβ accumulation [7, 33]. As reviewed by Tarashoff et al. [34], Aβ levels are affected by the deficits in endothelium function [34], enzymatic degradation [35–37], intramural periarterial drainage of Aβ [38], glymphatic paravascular clearance [7], lymphatic and immune clearance [10], and CSF absorption [39].

PET studies show that small molecular weight PET tau and amyloid tracers demonstrate rapid brain penetrance and clearance, with over 70% of the injected dose cleared from the brain by the study end [21, 40, 41]. We previously exploited this feature in our studies as a potential CSF clearance biomarker [16]. We reported in a small sample using THK5117 that ventricular CSF clearance is reduced in AD and inversely associated with brain Aβ levels. Now, we replicate our findings using another PET radiotracer, THK5351, and with a larger sample. Moreover, we observed for the first time correlations between CSF clearance and brain atrophy and cognitive functioning as related to AD.

MRI phase contrast studies have shown reduced CSF flow at the acqueduct in AD as compared to mild cognitive impairment (MCI) [42] and associated with cognitive deficits [43]. However, the phase contrast method measures pulsatile velocity rather than CSF flow and the results have been inconsistent [44, 45]. Intrathecal MR contrast studies directly show CSF flow and the glymphatic transport of Gd-DTPA through the brain ISF [46, 47]. While intrathecal contrast injection combined with dynamic contrast MRI appears to optimze the measurement of the CSF clearance, the intrathecal administration of contrast is invasive and it has limited application in clinical practice. Compared to these MRI measures, our intravenous dynamic PET measure is minimally invasive. Overall, the ventricle supplies the bulk of CSF to the brain [48] and therefore the rate of tracer removal from the ventricle is an attractive biomarker of the global CSF clearance.

With similar reasoning, Silverberg et al. used an invasive method with a ventricular catheter to test the hypothesis that impaired CSF dynamics were associated with AD [32]. They estimated the CSF production rate using intrathecal pressure changes before and after a volume of CSF was removed. However, this method is indirect and invasive, and a method that does not perturb the very system that is being measured would be preferable. More recently, in human, using a ^13^C_6_-leucine labelled Aβ and continuous lumbar spine CSF sampling, Bateman et al. observed that Aβ clearance was reduced 33% in AD but the Aβ production rate was unaffected [3, 6]. Consistent with Bateman et al., we observed a 18% reduction relative to NL group when CSF clearance was measured with ^18^F-THK5351 and 27% when measured with ^11^C-PiB PET.

### Impaired CSF clearance and brain amyloid

Our dynamic PET data suggest ventricular CSF clearance could be a useful biomarker to monitor CSF flow dysfunctions. Overall, our results are consistent with prior evidence showing that the increased residence time of Aβ contributes to its aggregation and fibrillization in the extracellular space [49]. We find reduced CSF clearance in AD for both THK5351 and PiB PET radiotracers, moderately strong associations with the extent of brain Aβ.

We observed that the two PET clearance measures were significantly correlated (r = 0.66, n = 24, p < 0.01). It is important to observe high correlation across tracers, even though the magnitude of brain binding is four-fold greater for PiB than for THK5351. This observation suggests that the VCSF-slope measure is independent of the global (specific and non-specific) tracer binding. Further highlighting the value of our observation, the clearance correlation with the amyloid burden was seen both in the total group and separately in the AD group.

However, the vCSF-slope measure does not inform us on specific pathways for clearing metabolic waste products from the brain parenchyma. Animal studies suggest that such pathways may involve: (a) perivascular CSF routes (for larger molecules) [47]; (b) intramural periarterial drainage [50]; (c) periaxonal/perineural routes [51] and (d) the vascular pathway via the BBB (for small size molecules) [1, 52, 53]. Aβ from the CSF is eliminated along perivascular pathways and enters the parenchyma along the pial-glial-basement membranes and this process is driven by vascular pulsations, which decrease in AD, potentially explaining the reduced clearance of the THK5351 and PiB PET radiotracers in the present study [50, 54, 55].

The ^11^C-PiB tracer, which also demonstrated utility as a CSF clearance agent, appears to be partially confounded by disease-related binding detected in the time frame used to estimate CSF clearance. We believe this is reflected in the greater estimated PiB clearance 27% vs 18% for THK5351, since some of the PiB tracer enters Aβ plaques in the 10–30 min time window. Overall, as compared with PiB, THK5351 has an advantage as a clearance agent. This supports the validity of the method and point towards a preference to the THK5351 for clearance estimations.

### Impaired CSF clearance is associated with decreased cognitive function

The CSF clearance measure and the brain amyloid binding were both associated with cognitive function. Intriguingly, in the subgroup analysis, the association of CSF clearance and cognitive function was significant in the NL group (r = − 0.83, n = 9, p < 0.01), while the correlation between brain amyloid binding and cognitive function was significant in the AD group (r = 0.58, n = 15, p < 0.05). Previous studies [56, 57] show that as many as 50% of ADAS-Cog component subscales demonstrate undesirable ceiling effects in subjects with mild or moderate AD. This may explain why the vCSF-slope fails to show a significant correlation with ADAS-Cog within our AD group. In spite of small sample size (n = 9) the correlation between vCSF and ADAS-cog was very strong in the control group. This finding is consistent with the observation that CSF clearance deficits may occur in presymptomatic stages of disease progression. Moreover, reduced clearance appear to be a risk factor for abnormal brain amyloid deposits. Here our small normal sample studied cross-sectionally, was at a disadvantage, as our healthy elderly volunteers did not demonstrate sufficient variation in amyloid binding for the correlations to reach significance. Most of the normal participants had very low binding levels. Nevertheless, these data suggest the hypothesis that CSF clearance measures have potential as a predictive biomarker at the pre Aβ lesion disease stage. However, in the absence of longitudinal data, this remains speculative. Of interest, a previous animal study showed clearance deficits prior to Aβ lesions [7].

### Confounds and study limitations

Ventricular CSF clearance could be confounded by both specific and non-specific binding of the tracer in the brain. However, our results suggest that over the time interval studied, clearance rates were independent of binding effects for THK5351. This is supported by the observation that ^18^F-THK5351, unlike ^11^C-PiB, did not show a global binding effect at the 10–30 min time interval. Additional evidence justifying that tracer brain binding has a small effect on CSF clearance rate is based on the high within-subject correlations for ^18^F-THK5351 and PiB (r = 0.66, p < 0.01), even though the tracers have different binding distribution volumes [58, 59]. Precise quantification would require using an 'inert' and permeable radiotracer that has no brain binding but undergoes rapid transit across the blood, CSF and the blood–brain barriers [60]. Future highly permeable radiotracers that do not exhibit brain binding may advance this work. Overall, the results suggest that brain tracer binding had minimal effect on vCSF-SLOPE for ^18^F-THK5351 and ^11^C-PiB.

Conventional compartmental PET models are designed for tracer binding and typically don’t include a CSF compartment or estimate its rate of clearance. More advanced compartmental models have been proposed [18], but have not been validated. New models for CSF dynamics that include tracer absorption recycling from blood and the exchange between the CSF and parenchymal interstitial space compartments are needed.

We evaluated several other possible confounds, including choroid plexus binding and partial-volume errors. Neither tracer showed choroid plexus binding that could potentially bias the ventricular clearance estimates. The ventricular partial volume error, due to contamination by proximity to brain, was minimized by individually sampling the ventricle 4 mm from the brain and with subsequent partial volume corrections [61]. Partial volume correction did not change the results. Another possible confound, the enlarged ventricular volume in AD may cause tracer dilution, thereby altering the clearance function. This was also tested and found not to affect the findings. Because of the MAO-B binding contamination, we determined the THK5351 tracer to be poorly suited for determining the cerebral tau burden. As a result, we don’t have reliable tau positivity information on study participants.

Overall, our cross-sectional findings are consistent with the hypothesis that CSF clearance is reduced in AD. Moreover, these data support a mechanism whereby the abnormal deposition of Aβ in the brain may be due to the failure of CSF clearance [3, 32]. However, a longitudinal sample is needed for estimating the directionality of the relationship between impaired clearance, brain Aβ deposits and the neurodegeneration we observed.

## Conclusions

Overall, the findings support the hypothesis that failed CSF clearance is a feature of AD that is related to Aβ deposition and to the pathology of AD. Longitudinal studies are needed to determine whether failed CSF clearance is a predictor of progressive amyloidosis or its consequence.

## Data Availability

The data that support the findings of this study are available from the corresponding authors upon reasonable request.

## Abbreviations

AD: Alzheimer’s disease
ADAS-cog: Alzheimer's Disease Assessment Scale–Cognitive Subscale
CDR: Clinical Dementia Rating Scale
CSF: Cerebrospinal fluid
MMSE: Mini-Mental State Exam
MRI: Magnetic Resonance Imaging
PET: Positron emission tomography
PiB: (11)C-labeled Pittsburgh Compound-B
SUV: Standardized uptake value
THK5117: 6-((3-Fluoro-2-hydroxy)propoxy)-2-(4-methylaminophenyl)quinoline
